# Excavation of diagnostic biomarkers and construction of prognostic model for clear cell renal cell carcinoma based on urine proteomics

**DOI:** 10.3389/fonc.2023.1170567

**Published:** 2023-05-16

**Authors:** Yiren Yang, Qingyang Pang, Meimian Hua, Zhao Huangfu, Rui Yan, Wenqiang Liu, Wei Zhang, Xiaolei Shi, Yifan Xu, Jiazi Shi

**Affiliations:** ^1^ Department of Urology, Changhai Hospital, Naval Medical University (Second Military Medical University), Shanghai, China; ^2^ Department of Urology, Changzheng Hospital, Naval Medical University (Second Military Medical University), Shanghai, China

**Keywords:** clear cell renal cell carcinoma, proteomics, urine, biomarker, prognosis

## Abstract

**Purpose:**

Clear cell renal cell carcinoma (ccRCC) is the most common pathology type in kidney cancer. However, the prognosis of advanced ccRCC is unsatisfactory. Thus, early diagnosis becomes one of the most important research priorities of ccRCC. However, currently available studies about ccRCC lack urine-related further studies. In this study, we applied proteomics to search urinary biomarkers to assist early diagnosis of ccRCC. In addition, we constructed a prognostic model to assist judge patients’ prognosis.

**Materials and methods:**

Urine which was used to perform 4D label-free quantitative proteomics was collected from 12 ccRCC patients and 11 non-tumor patients with no urinary system diseases. The urine of 12 patients with ccRCC confirmed by pathological examination after surgery was collected before operatoin. Bioinformatics analysis was used to describe the urinary proteomics landscape of these patients with ccRCC. The top ten proteins with the highest expression content were selected as the basis for subsequent validation. Urine from 46 ccRCC patients and 45 control patients were collected to use for verification by enzyme linked immunosorbent assay (ELISA). In order to assess the prognostic value of urine proteomics, a prognostic model was constructed by COX regression analysis on the intersection of RNA-sequencing data in The Cancer Genome Atlas (TCGA) database and our urine proteomic data.

**Results:**

133 proteins differentially expressed in the urinary samples were found and 85 proteins (Fold Change, FC>1.5) were identified up-regulated while 48 down-regulated (FC<0.5). Top 10 proteins including S100A14, PKHD1L1, FABP4, ITIH2, C3, C8G, C2, ATF6, ANGPTL6, F13B were performed ELISA to verify. The results showed that PKHD1L1, ANGPTL6, FABP4 and C3 were statistically significant (*P*<0.05). We performed multivariate logistic regression analysis and plotted a nomogram. Receiver operating characteristic (ROC) curve indicted that the diagnostic efficiency of combined indicators is satisfactory (Aare under curve, AUC=0.835). Furthermore, the prognostic value of the urine proteomics was explored through the intersection between urine proteomics and TCGA RNA-seq data. Thus, COX regression analysis showed that VSIG4, HLA-DRA, SERPINF1, and IGLV2-23 were statistically significant (*P*<0.05).

**Conclusion:**

Our study indicated that the application of urine proteomics to explore diagnostic biomarkers and to construct prognostic models of renal clear cell carcinoma is of certain clinical value. PKHD1L1, ANGPTL6, FABP4 and C3 can assist to diagnose ccRCC. The prognostic model constituted of VSIG4, HLA-DRA, SERPINF1, and IGLV2-23 can significantly predict the prognosis of ccRCC patients, but this still needs more clinical trials to verify.

## 1 Introduction

Renal cell carcinoma (RCC) is the most common kidney cancer, and data showed that about 17900 people died each year because of RCC (1). In addition, clear cell renal cell carcinoma (ccRCC) is the most common pathological type in RCC (2). However, the diagnosis of ccRCC is highly dependent on pathological diagnosis after surgery (3). Imaging methods have also been attempted by many researchers in assistant with diagnosis (4). The characteristics of computer tomography (CT) imaging and multiphasic MRI in ccRCC had already been summarized (5, 6). For example, previous study showed that diffusion kurtosis imaging (DKI) could be used to evaluate pathological grading of ccRCC (7). Although imaging characteristic indeed helps to diagnose ccRCC in some ways, it is not absolutely convincing.

Nowadays, liquid biopsy came into researchers’ view. The detection of exfoliated urinary cells has already become a routine diagnostic method in bladder cancers. It not only has the advantage of simple operation, but also has a high sensitivity. However, as the organ that produces urine, no urine biomarkers can be used in clinic for diagnosis nowadays. Due to the development of circulating tumor DNA (ctDNA) detection technology, this liquid biopsy method has been introduced into clinical practice (8). Several single-gene assays and multigene assays have been already approved, which signifies a point for the widespread use of liquid biopsy in the clinic (9). As for ccRCC, the recognition of molecular markers in serum and urine can be used for the screening, diagnosis and follow-up for patients (10). However, most researches concentrate in serum or urine metabolomics, microRNA and ctDNA (11–14). Our study applied proteomics to search urinary biomarkers to assist early diagnosis of ccRCC. We also investigated whether urine biomarkers can predict the prognosis of ccRCC, and developed a non-invasive and simple prognostic method.

## 2 Patients and methods

### 2.1 Urine proteomics for ccRCC patients and non-ccRCC patients

#### 2.1.1 Patients and methods

114 patients hospitalized in Changhai hospital (Shanghai, China) were included in the study from November 2021 to March 2022. This study was approved by the ethics committee of Changhai Hospital, Naval Medical University, and written informed consent was obtained from all patients. Urine from 23 patients were performed proteomics. Among them, 12 patients (Group R) were diagnosed with ccRCC pathologically after surgery and 11 non-ccRCC patients (Group C) which were defined as no cancers or other urinary system diseases patients were regarded as a control. In addition, urine from 91patients were collected to conduct enzyme linked immunosorbent assays (ELISA). 46 patients were assigned to Group R and 45 patients to Group C. All urine samples were collected in the morning before surgery. The detailed demographics are shown in **Tables 1**, **2**.

**Table 1:** Baseline data of patients for urine proteomics sequencing.

Variation	Group R(n=12)	Group C(n=11)	P-value
**Gender**			0.692
Male	9	9	
Female	3	2	
**Age**	59 ± 10.2	63.9 ± 6.7	0.191
**BMI**	24.1 ± 3.1	24.2 ± 1.9	0.882
**Smoking**			0.554
YES	3	4	
NO	9	7	
**Drinking**			0.855
YES	7	6	
NO	5	5	
**Diabetes**			0.949
YES	1	1	
NO	11	10	
**Hypertension**			0.855
YES	5	5	
NO	7	6	
**Hyperlipidemia**			0.538
YES	2	3	
NO	10	8	

**Table 2:** Baseline data of patients for ELISA validation.

Variation	Group R (n=46)	Group C (n=45)	P-value
**Gender**			0.143
Male	33	38	
Female	13	7	
**Age**	55.41 ± 11.82	59.11 ± 12.76	0.155
**BMI**	24.53 ± 3.38	24.11 ± 2.59	0.510
**Smoking**			0.074
YES	7	1	
NO	39	43	
**Drinking**			0.591
YES	7	5	
NO	39	39	
**Diabetes**			0.126
YES	8	3	
NO	38	41	
**Hypertension**			0.326
YES	17	12	
NO	29	32	
**Hyperlipidemia**			0.850
YES	8	7	
NO	38	37	

#### 2.1.2 Urine samples preparation

All urine samples were corrected in 4°C and then centrifugated at 4000× g for 15 minutes at 4°C. The supernatant was collected and stored at −80°C until analysis. Finally, the protein concentration was determined with BCA kit according to the manufacturer’s instructions.

#### 2.1.3 Liquid chromatography tandem-mass-spectrometry analysis

The tryptic peptides were dissolved in solvent A (0.1% formic acid, 2% acetonitrile/in water), directly loaded onto a home-made reversed-phase analytical column (25-cm length, 75/100 μm i.d.). Peptides were separated with a gradient from 6% to 24% solvent B (0.1% formic acid in acetonitrile) over 70 min, 24% to 35% in 14 min and climbing to 80% in 3 min then holding at 80% for the last 3 min, all at a constant flow rate of 450 nL/min on a nanoElute UHPLC system (Bruker Daltonics).

The peptides were subjected to capillary source followed by the timsTOF Pro (Bruker Daltonics) mass spectrometry. The electrospray voltage applied was 1.60 kV. Precursors and fragments were analyzed at the TOF detector, with a MS/MS scan range from 100 to 1700 m/z. The timsTOF Pro was operated in parallel accumulation serial fragmentation (PASEF) mode. Precursors with charge states 0 to 5 were selected for fragmentation, and 10 PASEF-MS/MS scans were acquired per cycle. The dynamic exclusion was set to 30 s.

#### 2.1.4 Database search

The resulting MS/MS data were processed using MaxQuant search engine (v.1.6.15.0). Tandem mass spectra were searched against the human SwissProt database (20422 entries) concatenated with reverse decoy database. Trypsin/P was specified as cleavage enzyme allowing up to 2 missing cleavages. The mass tolerance for precursor ions was set as 20 ppm in first search and 5 ppm in main search, and the mass tolerance for fragment ions was set as 0.02 Da. Carbamidomethyl on Cys was specified as fixed modification, and acetylation on protein N-terminal and oxidation on Met were specified as variable modifications. FDR was adjusted to < 1%.

### 2.2 Analyses of differentially expressed genes/proteins

The analyses of DEGs and DEPs were performed using the “limma” package in R statistical software, DEGs/DEPs were divided among two groups: Group R vs. Group C. DEGs/DEPs were defined by |log2 FC|>2 and P<0.05.

### 2.3 Measurement of candidate protein targets in urine using enzyme linked immunosorbent assay

After urine was collected, the particles and polymers were removed by centrifugation for 10 minutes after 3000rpm, and the supernatant was extracted. Remove the required strips from the aluminum foil bag after 20 min of room temperature balance, and put the remaining strips back at 4 °C after sealing with a self sealing bag. 50ul standard of different concentrations were added into the standard hole and 10ul samples into the sample holes, 40ul sample diluent into holes while blank holes are not added. Except the blank hole, each hole in the standard hole and sample hole is added with 100ul horseradish peroxidase (HRP) labeled antibody. Seal the reaction hole with sealing plate film, and incubate it in 37°C water bath or thermostat for 60min. Add 50ul substrates A and B into each hole. Incubate in dark at 37°C for 15 min. Add 50ul terminating liquid into each hole. Measure the OD value of each hole at the wavelength of 450nm within 15min.

### 2.4 Bioinformatics analysis

Statistical analysis and visualization of differential genes expression in tumor and normal tissues were completed using R (version 3.6.3) and graphs were produced using ggplot2 package in R. The data was downloaded from TCGA (https://portal.gdc.cancer.gov/) RNAseq data in level 3 HTSeq FPKM format in renal clear cell carcinoma(KIRC) project. Single gene survival analysis was performed using Kaplan-Meier (KM) method on GEPIA2 (GEPIA 2 (cancer-pku.cn)). Single gene immune microenvironment analysis and immune cell infiltration analysis are completed through SangerBox3.0 (www.sangerbox.com). Volcano plot, Venn, Multivariate cox regression analysis and Risk Score were also completed using R with ggplot2.

### 2.5 Development of diagnostic and prognostic nomogram model for survival

The diagnostic and prognostic nomogram models were developed in R software for Windows version 3.6.3 using the rms package (6.2-0 version) and survival package (3.2-10 version). The data about diagnostic model were collected from the patients who were performed ELSIA. While the prognostic data were collected from TCGA (https://portal.gdc.cancer.gov/) RNA-seq data in level 3 HTSeq-FPKM format in STAD (renal clear cell carcinoma) project and the RNA-seq data in FPKM (Fragments Per Kilobase per Million) format is converted into TPM (scripts per million reads) format and converted into log2. The difference in survival between the two groups was shown with Kaplan-Meier curves.

### 2.6 Statistical data analysis

Statistical analysis was carried out in R software for Windows version 3.6.3 and GraphPad Prism version 6.0 (GraphPad Software, Inc.; San Diego, CA, USA). The Shapiro normality test and visual inspection of the histograms were used to assess the data distribution. Differences were considered statistically significant if p-value was ≤ 0.05. The clinical parameters are expressed as mean ± standard deviation (SD).

### 2.7 The experimental strategy

**Figure 1:**
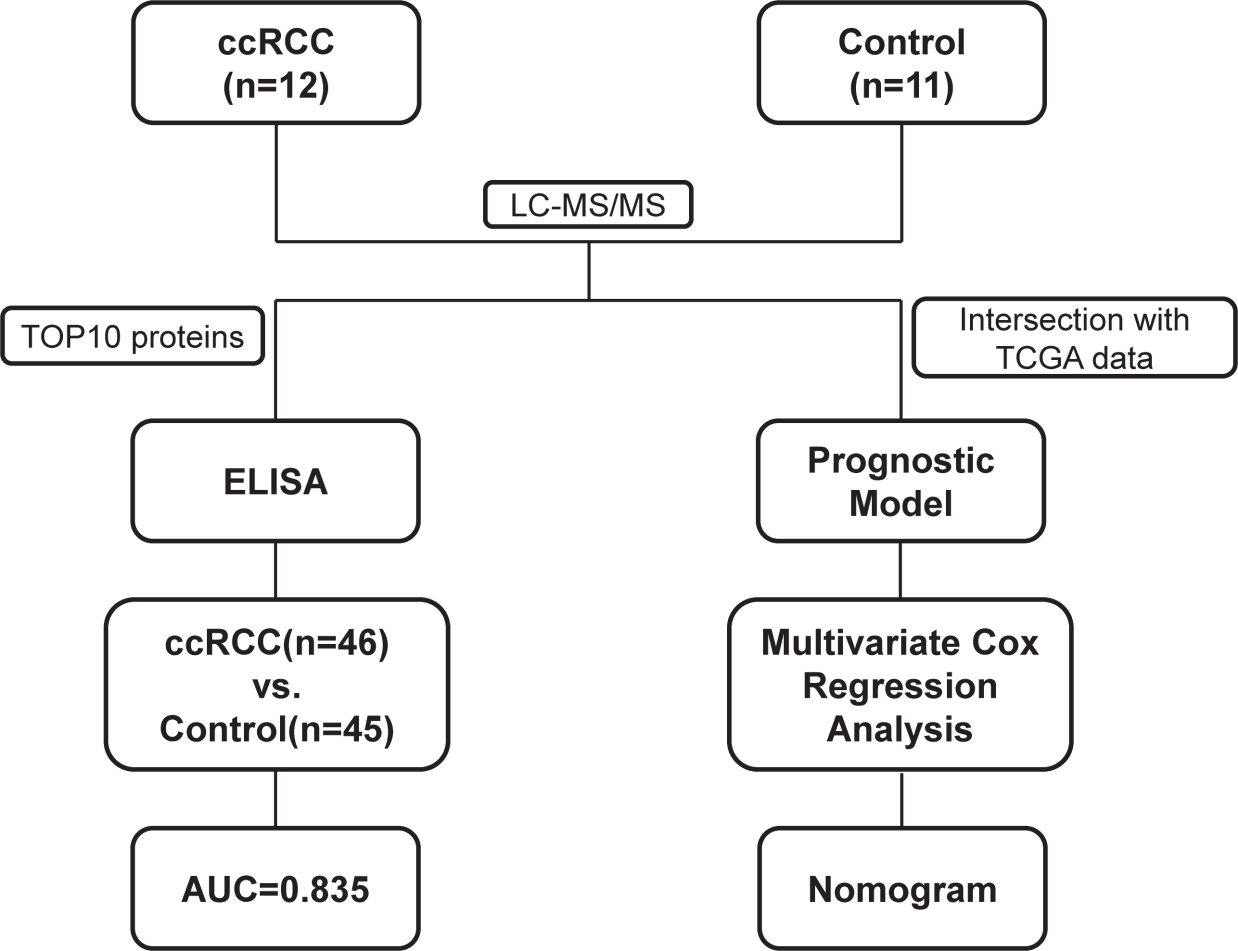
Flowchart of proteomic analyses in clear cell renal cell carcinoma(ccRCC) patients and control patients.

The major aim of our study is to explore urine biomarkers and construct a prognostic model to assist diagnosis and prediction. The experimental strategy is shown in **Figure 1**. We performed a urine proteomics to select diagnostic biomarkers *via* comparing Group R and Group C. Then we chose the top 10 proteins to verify using enzyme linked immunosorbent assay (ELISA). At the same time, we took the intersection of our urine proteomics data and KIRC RNA-seq data in TCGA to construct a prognostic model.

## 3 Results

### 3.1 Urine proteomics profile of ccRCC patients and non-ccRCC patients

**Figure 2:**
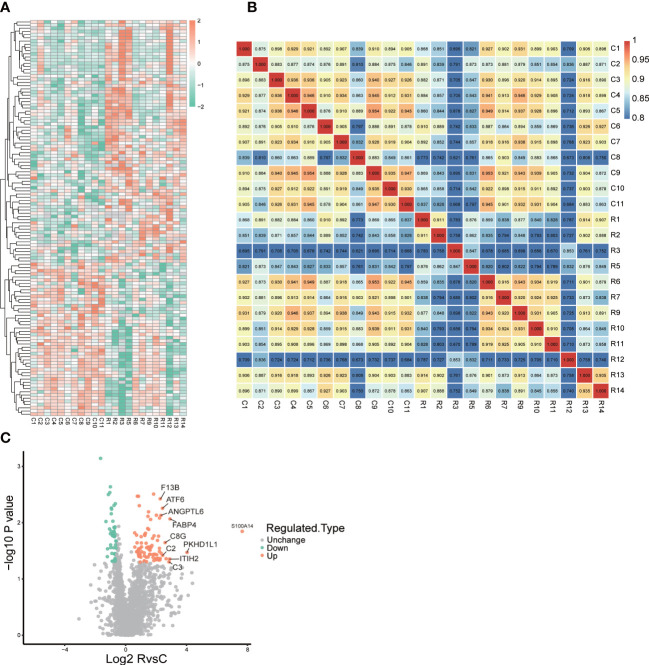
Landscape of proteomics in ccRCC patients and control patients. **(A)** heatmap of all different expression proteins(DEPs) **(B)** The heatmap drawn by Pearson correlation coefficient between two pairs of all samples **(C)** volcano plot of all DEPs, and top10 up-regulated proteins were remarked in the plot.

We collected urine samples from 12 ccRCC patients and 11 non-ccRCC patients, and performed 4D label-free quantitative proteomics. 133 proteins differentially expressed in the urinary samples were found and 85 proteins (Fold Change, FC>1.5) were identified up-regulated while 48 down-regulated (FC<0.5). Besides, we present a heat map of all the differentially expressed genes in **Figure 2A**. Correlation analysis showed the heterogeneity of urine samples (**Figure 2B**). In combination with **Figures 2A, B**, it can be seen that urine proteins are highly heterogeneous. But there is still plenty of significant information to be minded. So we selected the TOP10 proteins: S100A14, PKHD1L1, FABP4, ITIH2, C3, C8G, C2, ATF6, ANGPTL6 and F13B as ccRCC potential diagnostic biomarkers (**Figure 2C**).

### 3.2 PKHD1L1, FABP4, C3 and ANGPTL6 can be utilized to diagnose ccRCC

**Figure 3:**
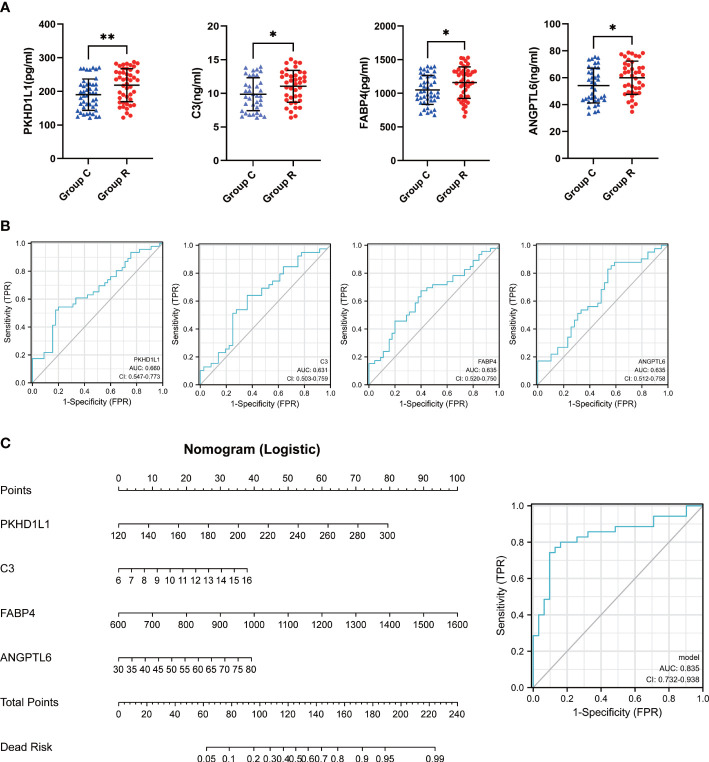
Construction of diagnostic model. **(A)** scatter plots for Elisa results. **(B)** ROC curves for the individual diagnostic sensitivity of PKHL1L1, C3, FABP4, ANGPTL6. **(C)** nomogram and ROC curves for combined PKHL1L1, C3, FABP4, ANGPTL6. *p < 0.05, **p < 0.01.

In order to verify whether TOP10 proteins can work as diagnostic biomarkers for ccRCC, we collected urine from 46 ccRCC patients and 45 non-ccRCC patients and performed ELISA to test it (**Figure 3A**). As a result, the difference between Group R and Group C of PKHD1L1, FABP4, C3 and ANGPTL6 has statistical significance. In addition, as we expected, the content of these four proteins in the urine of ccRCC patients is indeed higher than that of non-ccRCC patients. However, S100A14, ITIH2, C8G, C2, ATF6, and F13B have no statistical significance between two groups (**Figure S1A**). Then we found that the diagnostic efficiency of PKHD1L1, FABP4, C3 and ANGPTL6 seems to be dissatisfactory individually (**Figure 3B**). However, we performed multivariate logistic regression analysis and plotted a nomogram (**Figure 3C**). Most importantly, the diagnostic efficiency is raised to 0.835 using the nomogram to diagnose ccRCC.

### 3.3 A prognostic model containing SERPINF1, HLA-DRA, VSIG4, and IGLV2-23

**Figure 4:**
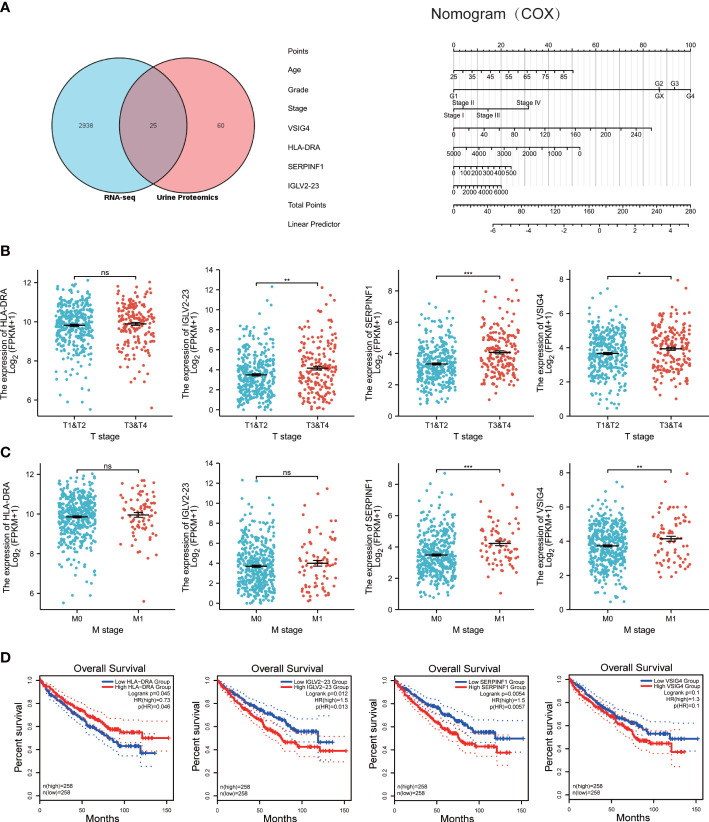
Construction of prognostic model, and prognosis for HLA-DRA, IGLV2-23, SERPINF1 and VSIG4. **(A)** left: venn diagram of TCGA RNA-seq data and Urine proteomics. Right: nomogram for the prognostic model of SERPINF1, HLA-DRA, VSIG4 and IGLV2-23. **(B)** scatter plots of the relation between T stage and expression of HLA-DRA, IGLV2-23, SERPINF1 and VSIG4. **(C)** scatter plots of the relation between M stage and expression of HLA-DRA, IGLV2-23, SERPINF1 and VSIG4. **(D)** Kaplan-Meier curves of overall survival(OS) for HLA-DRA, IGLV2-23, SERPINF1 and VSIG4. ns: p>0.05, *p < 0.05, **p < 0.01, ***p < 0.001.

What’s more, we are highly interested in whether urine protein can guide the prognosis of ccRCC patients. Thus, we downloaded TCGA KIRC RNA-seq data, and took the intersection of both datasets (**Figure 4A**). 25 genes appear in the intersection and we did COX regression analysis on them. As a result, SERPINF1, HLA-DRA, VSIG4 and IGLV2-23 have statistical significance in COX regression analysis and we combined these four molecules with the patients’ age, pathological grade and pathological stage to build a nomogram model to make it visualized (**Figure 4B**).

We further explored the relation between T and M stage and the expression of SERPINF1, HLA-DRA, VSIG4 and IGLV2-23. As the **Figures 4B, C** showed, the expression of HLA-DRA is not related to T stage and M stage. The expression of IGLV2-23, SERPINF1 and VSIG4 is relevant to T stage and except IGLV2-23, the others are also bound up with M stage. As for OS, high expression of HLA-DRA, IGLV2-23 and SERPINF1 indicated worse prognosis but no differences can be found between high and low VSIG4 group (**Figure 4D**). Although there are no differences between T or M stage and the expression of HLA-DRA, statistical significance exists in the prognosis of HLA-DRA.

**Figure 5:**
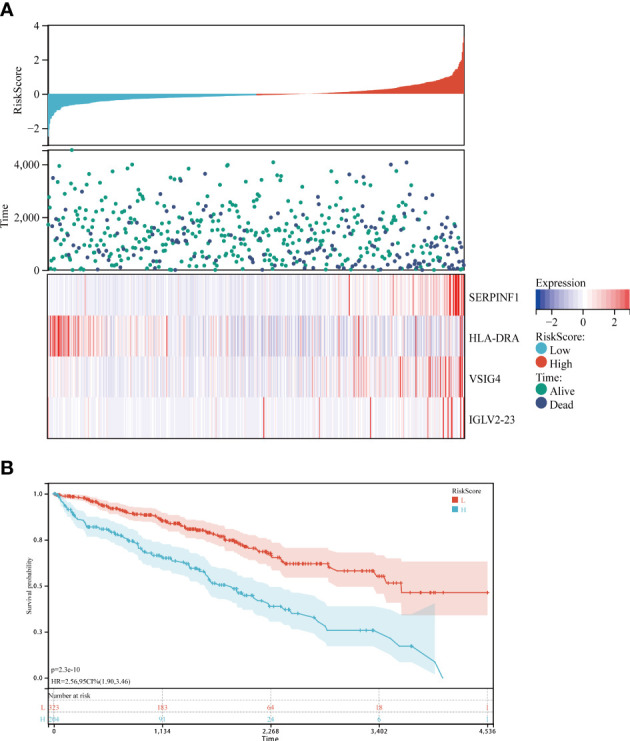
Predictive efficiency of the prognostic model. **(A)** Risk factor plot of high-risk group and low-risk group. **(B)** K-M curves of high-risk group and low-risk group.

Afterwards, we calculated the survival risk score of each case according to the expression level of these four genes, and then divided the patients into high-risk group and low-risk group. (**Figure 5A**). Risk factor diagram indicted that higher expression of SRPINF1, VSIG4 and IGLV2-23 is correlated with higher risk scores. However, higher expression of HLA-DRA is related to lower risk scores. Exactly as we expected, high risk score group has worse prognosis, which means those patients with high risk scores tend to have shorter survival time (**Figure 5B**).

## 4 Discussion

### 4.1 The importance of early diagnosis of RCC and limitations of current diagnostic methods

The pathological type of most renal cell carcinomas is renal clear cell carcinoma (2). The prognosis of patients with renal cell carcinoma is closely related to the tumor stage at initial diagnosis. The 5-year cancer-specific survival rate for treated patients with localized renal cancer is 95% (15). However, patients with advanced renal cell carcinoma usually have a poor prognosis. Targeted therapy is the most widely used method for patients with advanced renal cell carcinoma, such as sorafenib, sunitinib, pezopanib and cabozantinib (16–19).. However, many patients did not respond to the initial treatment, or developed secondary drug resistance after about 1 year of treatment (20). At present, the most advanced therapy for advanced renal cell carcinoma, which combined targeted therapy with immunotherapy, has achieved good results, but it is still not ideal. Therefore, early detection of renal cancer is very important, it can enable patients to get early treatment and obtain the opportunity to cure the tumor.

RCC often presents as an occult tumor. More than 50% of kidney cancers are discovered by accident because of the absence of clinical symptoms and abnormal signs (21). At present, the examinations for kidney cancer mainly include ultrasound, computed tomography (CT) scan and magnetic resonance imaging (MRI). Ultrasound examination is a convenient and inexpensive examination method, can be used as a primary screening method. However, the detection rate of RCC may be affected by the skill level of the sonographer. CT and MRI can accurately identify tumors, but are expensive and not suitable for routine screening in RCC. Moreover, some patients, such as patients with renal insufficiency, pregnant women and patients with allergy to contrast material, should not undergo CT examination. Therefore, there is an urgent need for simple and accurate biomarkers for early screening of RCC. The kidney is the origin organ of urine formation and has great influence on the composition of urine. And urine is a body fluid that can be collected in a completely noninvasive way.

### 4.2 Urine markers of RCC

In a 2004 study, proteomic tests were performed on the urine of a kidney cancer patient before and after surgery (22). Researchers found that the precursors of the mannan-binding lectin serine protease 2 and the kininogen were reduced after nephrectomy. Among them, the decline of the kininogen, which mediates inflammation and improves vascular permeability, was particularly significant. Bosso et al. (23) performed proteomic test and found three peaks that distinguished the urine of patients with ccRCC from that of normal control patients. The specificity and sensitivity of the three peaks in the diagnosis of renal cancer were 100% and 95%, respectively. Liu et al. (14) analyzed the urine metabolites of 100 patients with RCC, 34 patients with benign renal tumor and 129 normal controls. The researchers found that the combination of the nine metabolites had a good predictive ability of RCC, with the AUC reaching 0.905 in the training set and 0.885 in the validation set. In addition, the nine-metabolite group also played a role in distinguishing RCC from renal benign tumors, with an AUC of 0.816. Among them, N-formylkynurenine showed the highest diagnostic efficacy for RCC, with an AUC of 0.808. Liquid urine biopsy can be used not only for early screening of kidney cancer, but also for predicting the recurrence of kidney cancer after surgery. Morozumi et al. (24) identified five metabolites (lactic acid, glycine, 2- hydroxyglutarate, succinic acid, and kynurenic acid) that were used to predict the recurrence of kidney cancer by detecting urine in patients with renal cancer. The AUC, sensitivity and specificity of the model were 0.894, 88.9% and 88.0%, respectively. Patients were classified by this model into the low-risk and high-risk group for recurrence. Recurrence-free survival was found to be significantly reduced in the high-risk group.

### 4.3 Diagnostic model and prognostic model of ccRCC based on urine proteomics

Despite these studies, there is still no recognized urine marker for effective screening of ccRCC (25). Therefore, we conducted proteomic sequencing using the urine of ccRCC patients and non-tumor patients. According to the sequencing results, the diagnostic model and prognosis model of ccRCC were constructed based on urine proteomics.

The diagnostic model was composed of PKHD1L1, FABP4, C3 and ANGPTL6. ANGPTL6 is one of the seven members of ANGPTLs. ANGPTL6 was first discovered in 2003 and is considered to be an angiogenic factor involved in epidermal proliferation and wound healing (26, 27). ANGPTL6 is also associated with metabolism. The polymorphism of ANGPTL6 may be closely related to metabolic syndrome (28). The correlation between ANGPTL6 and tumor has been reported. Hu et al. found that high ANGPTL6 is associated with high risk and poor prognosis of HCC. And ANGPTL6 was a biomarker for diagnosis and prognosis of HCC (29). High levels of ANGPTL6 were associated with poor prognosis (30). This study confirmed for the first time that urine ANGPTL6 protein was a diagnostic marker of ccRCC. However, the expression of ANGPTL6 in normal renal tissue was higher than that in renal carcinoma tissue. In addition, high expression of ANGPTL6 in tumor tissues was associated with a poor prognosis. It was suggested that ANGPTL6 might have a complicated effect on tumor diagnosis and progression.

The subcellular localization and biological function of the PKHD1L1 gene product were unknown (31). The relationship between PKHD1L1 and cancer has been less reported. Zheng et al. (32) analyzed the sequencing results of 58 patients with papillary thyroid cancer, TCGA data and cell assay results. Researchers found that PKHD1L1 might be a tumor suppressor gene associated with papillary thyroid cancer and might be a potential therapeutic target in the future. In this study, it was found that PKHD1L1 was significantly increased in the urine of ccRCC and was one of the genes in the diagnostic model. However, similar to ANGPTL6, the expression of PKHD1L1 in normal kidney tissues was higher than that in renal cancer tissues, and the high expression of PKHD1L1 also predicted a poor prognosis.

The complement system refers to a class of serum proteins that play an important role in inflammation. Among them, C3 is abundant in human body and is associated with tumor. C3 has been found to promote neutrophil recruitment and the formation of neutrophil extracellular traps (NETs), which promote cancer cell metastasis to the lung (33). Ye et al. (34) found that complement C3 deficiency in patients with gastric cancer was associated with poor short-term prognosis and poor long-term prognosis. Yuan et al. (35) found that C3 deposition in gastric cancer tissues was higher than that in paired normal tissues, and was positively correlated with TNM staging of tumors. High C3 deposition was an independent risk factor for poor 5-year survival. In this study, C3 in urine was found to be a factor in the diagnostic model of ccRCC. The expression of C3 in ccRCC was higher than that in normal renal tissue. High C3 is associated with poor prognosis. This suggests that C3 also plays a cancer-promoting role in ccRCC.

FABP4 was mainly expressed in adipocytes and macrophages and was involved in obesity-induced insulin resistance (36–38). FABP4 can regulate tumor growth, migration and invasion by affecting metabolism. FABP4 is closely related to tumors. Inhibition of FABP4 can induce apoptosis of breast cancer cells (39). FABP4 overexpression can affect the metabolism of colon cancer cells and promote their migration and invasion (40). In this study, we found that urine FABP4 protein could be used as an important component of the ccRCC diagnostic model, although there was no difference in FABP4 expression between tumor tissue and normal kidney tissue. It was suggested that the release of FABP4 in kidney cancer tissues might be different from that in normal kidney tissues, which might be one of the potential characteristics of renal cancer cells.

We further explored the factors that may represent the prognosis of ccRCC patients in urine and constructed a model formed by SERPINF1, HLA-DRA, VSIG4 and IGLV2-23. SERPINF1 is the pathogenic gene of Osteogenesis imperfecta type VI. Previous studies have found that SERPINF1 high expression represents a poor prognosis for ccRCC (41). Human leukocyte antigen (HLA)-DR alpha chain (DRA) is a major histocompatibility complex (MHC) Class II antigen that plays an important role in immunity. Previous studies have confirmed that HLA-DRA is associated with the prognosis of ccRCC and bladder cancer (42, 43). V-set and immunoglobulin domain containing 4 (VSIG4) is a type I transmembrane receptor that plays an important role in the maintenance of immune homeostasis, but also promotes cancer progression (44). And few studies have been conducted on the function of IGLV2-23.

The urine test is a completely noninvasive, promising liquid biopsy, especially for urinary diseases. According to the study, there were significant differences in urine protein between the patients with ccRCC and the control group. Diagnostic and prognostic models of ccRCC could be constructed based on differential proteins. Future studies with a larger sample size are needed for further confirmation, so as to make a relatively accurate preliminary screening of ccRCC and predict the prognosis of patients with ccRCC through urine examination.

## Data availability statement

The datasets presented in this study can be found in online repositories. The names of the repository/repositories and accession number(s) can be found in the article/**Supplementary Material**.

## Author contributions

YY, RY and MH conceived the present study. RY, ZH and QP performed the literature search and data analysis. The first draft of the manuscript was written by YY, ZH, YX and JS. WL, WZ, XS, YX, QP and JS critically revised the work. The revision of the review was performed by QP. All authors contributed to the article and approved the submitted version.
