# Supplementary figures and images for: Excavation of diagnostic biomarkers and construction of prognostic model for clear cell renal cell carcinoma based on urine proteomics
Source: Front Oncol. 2023 May 16;13:1170567. doi: 10.3389/fonc.2023.1170567 (PMC10228721; doi:10.3389/fonc.2023.1170567)

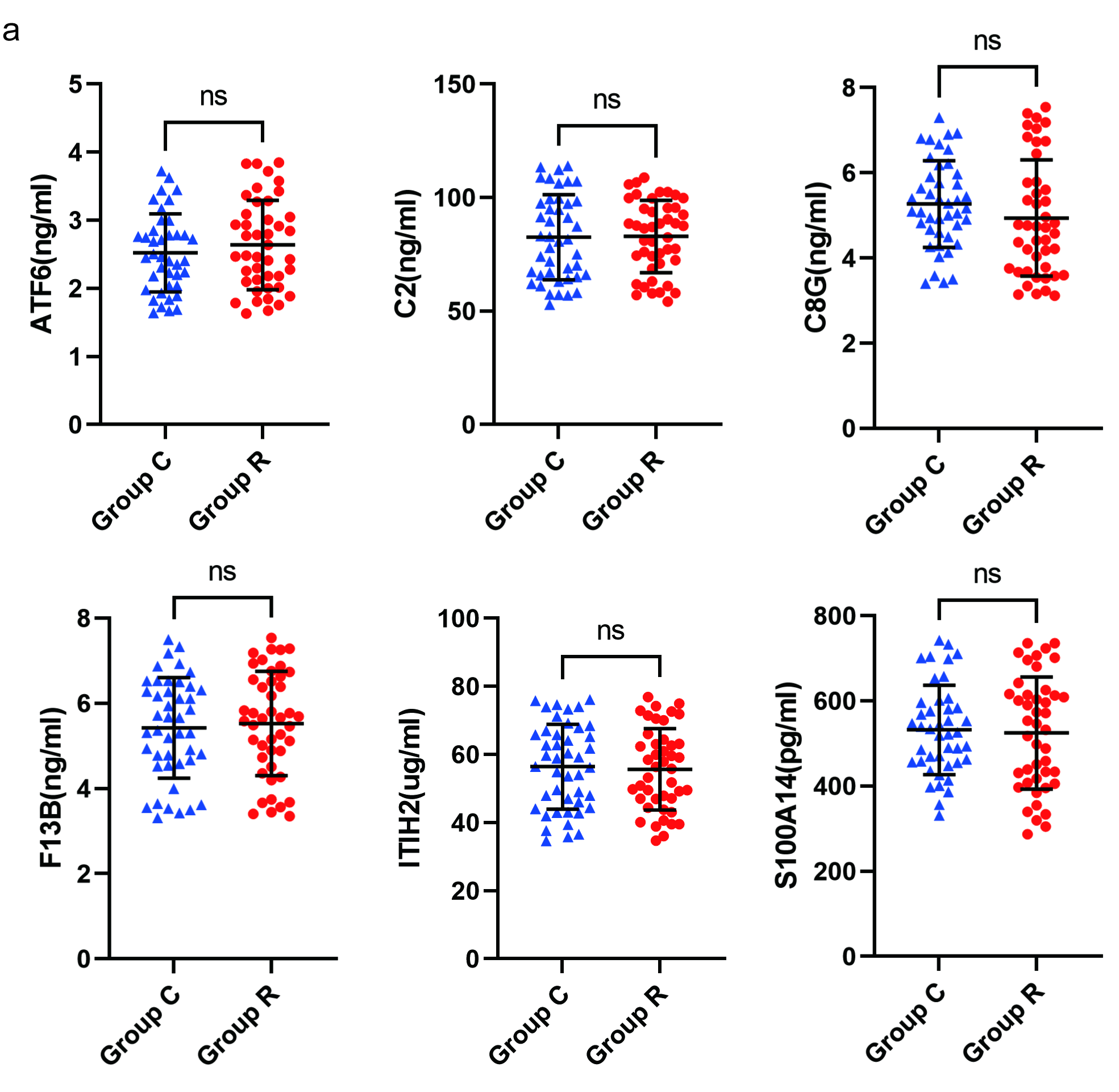

Supplement: Supplementary file 1 [file Image_1.tif]
